# PLD3 epigenetic changes in the hippocampus of Alzheimer’s disease

**DOI:** 10.1186/s13148-018-0547-3

**Published:** 2018-09-12

**Authors:** Idoia Blanco-Luquin, Miren Altuna, Javier Sánchez-Ruiz de Gordoa, Amaya Urdánoz-Casado, Miren Roldán, María Cámara, Victoria Zelaya, María Elena Erro, Carmen Echavarri, Maite Mendioroz

**Affiliations:** 1 Neuroepigenetics Laboratory-Navarrabiomed, Complejo Hospitalario de Navarra, Universidad Pública de Navarra (UPNA), IdiSNA (Navarra Institute for Health Research), C/ Irunlarrea, 3, 31008 Pamplona, Navarra Spain; 2 Department of Neurology, Complejo Hospitalario de Navarra- IdiSNA (Navarra Institute for Health Research), C/ Irunlarrea, 3, 31008 Pamplona, Navarra Spain; 3 Department of Pathology, Complejo Hospitalario de Navarra- IdiSNA (Navarra Institute for Health Research), 31008 Pamplona, Navarra Spain; 4 Hospital Psicogeriátrico Josefina Arregui, 31800 Alsasua, Navarra Spain

**Keywords:** PLD3, Alzheimer’s disease, Epigenetics, DNA methylation, Gene and protein expression, Hippocampus, APP, Lysosome

## Abstract

**Background:**

Whole-exome sequencing has revealed a rare missense variant in *PLD3* gene (rs145999145) to be associated with late onset Alzheimer’s disease (AD). Nevertheless, the association remains controversial and little is known about the role of *PLD3* in AD. Interestingly, *PLD3* encodes a phospholipase that may be involved in amyloid precursor protein (APP) processing. Our aim was to gain insight into the epigenetic mechanisms regulating *PLD3* gene expression in the human hippocampus affected by AD.

**Results:**

We assessed *PLD3* mRNA expression by qPCR and protein levels by Western blot in frozen hippocampal samples from a cohort of neuropathologically confirmed pure AD cases and controls. Next, we profiled DNA methylation at cytosine-phosphate-guanine dinucleotide (CpG) site resolution by pyrosequencing and further validated results by bisulfite cloning sequencing in two promoter regions of the *PLD3* gene. A 1.67-fold decrease in *PLD3* mRNA levels (*p* value < 0.001) was observed in the hippocampus of AD cases compared to controls, and a slight decrease was also found by Western blot at protein level. Moreover, *PLD3* mRNA levels inversely correlated with the average area of β-amyloid burden (tau-b = − 0,331; *p* value < 0.01) in the hippocampus. A differentially methylated region was identified within the alternative promoter of *PLD3* gene showing higher DNA methylation levels in the AD hippocampus compared to controls (21.7 ± 4.7% vs. 18.3 ± 4.8%; *p* value < 0.05).

**Conclusions:**

*PLD3* gene is downregulated in the human hippocampus in AD cases compared to controls. Altered epigenetic mechanisms, such as differential DNA methylation within an alternative promoter of *PLD3* gene, may be involved in the pathological processes of AD. Moreover, *PLD3* mRNA expression inversely correlates with hippocampal β-amyloid burden, which adds evidence to the hypothesis that PLD3 protein may contribute to AD development by modifying APP processing.

**Electronic supplementary material:**

The online version of this article (10.1186/s13148-018-0547-3) contains supplementary material, which is available to authorized users.

## Background

Alzheimer’s disease (AD) is a genetically complex process where ε4 allele of the *APOE* gene (APOE4) is by far the best-established genetic susceptibility risk factor. In addition, genome-wide association studies have revealed a considerable number of small-effect common variants in genes related to AD [1–3]. However, those variants do not explain the full heritability of this disease. More recently, novel sequencing technologies are enabling the identification of other rare genetic variants that could potentially contribute to the development of sporadic AD. Notable recent discoveries in this area include rare disease variants in *TREM2*, *UNC5C*, *AKAP9*, *TM2D3*, *ADAM10*, and *PLD3* genes [2, 4, 5].

*PLD3* (phospholipase D family, member 3) (OMIM * 615698) gene is located at chromosome 19q13.2 and encodes a lysosomal protein that belongs to the phospholipase D (PLD) superfamily, which catalyzes the hydrolysis of membrane phospholipids. However, PLD3 catalytic function has not yet been demonstrated [6, 7]. *PLD3* gene is highly expressed in the brain of healthy controls, particularly in several brain regions vulnerable to AD pathology, such as frontal, temporal, and occipital cortices and hippocampus, but reduced in neurons from AD brains [3, 8]. Nevertheless, little is known on the regulation, the function, and the involvement of *PLD3* in AD pathogenesis.

Interestingly, controversial association exists about this gene conferring increased risk for the development of AD. Cruchaga et al. performed whole-exome sequencing on AD patients and identified a rare missense variant (rs145999145) in exon 7 of the *PLD3* gene which resulted in a val232-to-met (V232M) substitution [8]. Their results revealed that carriers of the *PLD3* coding variant had a twofold increased risk for late onset AD. Moreover, they showed that PLD3 influences amyloid precursor protein (APP) processing, acting as a negative regulator, since PLD3 overexpression in cultured neuroblastoma cells correlated with lower intracellular APP, extracellular Aβ42, and Aβ40 levels and that PLD3 protein could be co-immunoprecipitated with APP. In that regard, Satoh et al. showed an accumulation of PLD3 on neuritic plaques in AD brains and suggested a key role for PLD3 in the pathological processes of AD [9].

Other authors confirmed that *PLD3* gene variant V232M was associated with AD risk and significantly lower cognitive function [10] providing a systematic view of the involvement of *PLD3* in AD at genetic, mRNA, and protein level expression. However, additional studies were not able to define an essential role of *PLD3* rare variants in AD [11], neither to support an important contribution of *PLD3* rare variants in the etiology of AD, given the high variability of the frequency of *PLD3* Val232Met variant across populations [12]. Indeed, follow-up studies have questioned the role of *PLD3* rare variants in AD, obtaining negative replication data [13–15] and suggesting a more complex role of *PLD3* in the etiology of the disease.

Keeping in mind the results mentioned above, we wanted to gain insight into the epigenetic mechanisms regulating *PLD3* expression in order to add evidence to the potential contribution of *PLD3* to AD. Further knowledge on these mechanisms may provide opportunities for new AD therapeutic strategies. Here, we profiled *PLD3* gene expression and methylation in the human hippocampus, one of the most vulnerable brain regions to AD. To that end, we selected a cohort of neuropathologically defined “pure” AD cases and controls to measure hippocampal *PLD3* expression by quantitative PCR and Western blot. Next, we explored the correlation of *PLD3* expression with AD neuropathological changes. Finally, DNA methylation levels at two distinct promoter regions of the *PLD3* gene were assessed by pyrosequencing and bisulfite cloning sequencing.

## Methods

### Human hippocampal samples and neuropathological examination

Brain hippocampal samples from 30 AD patients and 12 controls were provided by Navarrabiomed Brain Bank. After death, half brain specimens from donors were cryopreserved at − 80 °C. Neuropathological examination was completed following the usual recommendations [16] and according to the updated National Institute on Aging-Alzheimer’s Association guidelines [17]. Assessment of β-amyloid deposition was carried out by immunohistochemical staining of paraffin-embedded sections (3–5 μm thick) with a mouse monoclonal (S6F/3D) anti β-amyloid antibody (Leica Biosystems Newcastle Ltd., Newcastle upon Tyne, UK). Evaluation of neurofibrillary pathology was performed with a mouse monoclonal antibody anti-human PHF-TAU, clone AT-8, (Tau AT8) (Innogenetics, Gent, Belgium), which identifies hyperphosphorylated tau (p-tau) [18]. The reaction product was visualized using an automated slide immunostainer (Leica Bond Max) with Bond Polymer Refine Detection (Leica Biosystems, Newcastle Ltd.).

To avoid spurious findings related to multiprotein deposits, “pure” AD cases with deposits of only p-tau and β-amyloid were eligible for the study and controls were free of any pathological protein aggregate. This approach maximizes chances of finding true associations with AD, even though reducing the final sample size. A summary of characteristics of subjects included in this study is shown in Additional file 1: Table S1. AD subjects were older than controls (82.3 ± 11.3 versus 50.7 ± 21.5; *p* value < 0.01), and no differences were found regarding gender (*p* value = 0.087). The postmortem interval (PMI) were not significantly different between groups (8.2 ± 4.4 h in the control group versus 7.9 ± 7.1 h in the AD group; *p* value = 0.91).

### *PLD3* mRNA expression analysis by RT-qPCR

Total RNA was isolated from hippocampal homogenates using RNeasy Lipid Tissue Mini kit (QIAGEN, Redwood City, CA, USA), following the manufacturer’s instructions. Genomic DNA was removed with recombinant DNase (TURBO DNA-free™ Kit, Ambion, Inc., Austin, TX, USA). RNA integrity was checked by 1.25% agarose gel electrophoresis under denaturing conditions. Concentration and purity of RNA were both evaluated with NanoDrop spectrophotometer. Only RNA samples showing a minimum quality index (260 nm/280 nm absorbance ratios between 1.8 and 2.2 and 260 nm/230 nm absorbance ratios higher than 1.8) were included in the study. Complementary DNA (cDNA) was reverse transcribed from 1500 ng total RNA with SuperScript® III First-Strand Synthesis Reverse Transcriptase (Invitrogen, Carlsbad, CA, USA) after priming with oligo-d (T) and random primers. RT-qPCR reactions were performed in triplicate with Power SYBR Green PCR Master Mix (Invitrogen, Carlsbad, CA, USA) in a QuantStudio 12K Flex Real-Time PCR System (Applied Biosystems, Foster City, CA, USA) and repeated twice within independent cDNA sets. Sequences of primer pair were designed using Real Time PCR tool (IDT, Coralville, IA, USA) and are listed in Additional file 1: Table S2. Relative expression level of *PLD3* mRNA in a particular sample was calculated as previously described [19] and *ACTB* gene was used as the reference gene to normalize expression values.

### *PLD3* protein expression analysis by Western blot

Human hippocampus tissue from patients and control samples was lysed with 100 μL lysis buffer containing urea, thiourea, and DTT. After centrifugation at 35.000 rpm for 1 h at 15 °C, extracted proteins were quantified following the Bradford-Protein Assay (Bio-Rad, Hercules, CA, USA) by using a spectrophotometer.

Next, 5 μg of protein per sample were resolved in 4–20% Criterion TGX stain-free gels (Bio-Rad) and electrophoretically transferred onto nitrocellulose membranes using a Trans-blot Turbo transfer system (25 V, 7 min) (Bio-Rad). Equal loading of the gel was assessed by stain-free digitalization and by Ponceau staining. Membranes were probed with rabbit anti-human PLD3 primary antibody (Sigma-Aldrich; 1:250) in 5% nonfat milk and incubated with peroxidase-conjugated anti-rabbit secondary antibody (Cell Signaling; 1:2000). Immunoblots were then visualized by exposure to an enhanced chemiluminescence Clarity Western ECL Substrate (Bio-Rad) using a ChemidocMP Imaging System (Bio-Rad). Expression levels of PLD3 were standardized by the corresponding band intensity of GAPDH (Calbiochem; 1:10000).

### Quantitative assessment of β-amyloid and p-tau deposits in hippocampal samples

In order to quantitatively assess the β-amyloid and p-tau burden for further statistical analysis, we applied a method to quantify protein deposits, as described in detail elsewhere [20]. In brief, hippocampal sections were examined after performing immunostaining with anti β-amyloid and anti p-tau antibodies. Focal deposit of β-amyloid, including neuritic, immature, and compact plaques [21], was analyzed with the ImageJ software. Moreover, β-amyloid plaque count, referred to as amyloid plaque score (APS), was measured. Finally, p-tau deposit was also analyzed with ImageJ software in order to obtain an average quantitative measure of the global p-tau deposit for each section.

### *PLD3* methylation measurement by pyrosequencing

Genomic DNA was isolated from frozen hippocampal tissue by phenol-chloroform method [22]. Next, 500 ng of genomic DNA was bisulfite converted using the EpiTect Bisulfite Kit (Qiagen, Redwood City, CA, USA) according to the manufacturer’s protocol. Primers to amplify and sequence two promoter regions of *PLD3* were designed with PyroMark Assay Design version 2.0.1.15 (Qiagen) (Additional file 1: Table S2), and PCR reactions were carried out on a VeritiTM Thermal Cycler (Applied Biosystems, Foster City, CA, USA). Next, 20 μl of biotinylated PCR product was immobilized using streptavidin-coated sepharose beads (GE Healthcare Life Sciences, Piscataway, NJ, USA) and 0.3 μM sequencing primer was annealed to purified DNA strands. Pyrosequencing was performed using the PyroMark Gold Q96 reagents (Qiagen) on a PyroMark™ Q96 ID System (Qiagen). For each particular cytosine-phosphate-guanine dinucleotide (CpG), methylation levels were expressed as percentage of methylated cytosines over the sum of total cytosines. Unmethylated and methylated DNA samples (EpiTect PCR Control DNA Set, Qiagen) were used as controls for the pyrosequencing reaction.

### *PLD3* methylation validations by bisulfite cloning sequencing

Bisulfite-converted genomic DNA was used to validate pyrosequencing results. Primer pair sequences were designed by MethPrimer [23] and are listed in Additional file 1: Table S2. PCR products were cloned using the TopoTA Cloning System (Invitrogen, Carlsbad, CA, USA), and a minimum of 10–12 independent clones were sequenced for each examined subject and region. Methylation graphs were obtained with QUMA software [24].

### Statistical data analysis

Statistical analysis was performed with SPSS 21.0 (IBM, Inc., USA). Before performing differential analysis, we checked that all continuous variables showed a normal distribution, as per one-sample Kolgomorov-Smirnov test and the normal quantil-quantil (QQ) plots. Data represents the mean ± standard deviation (SD). Significance level was set at *p* value < 0.05. Statistical significance for *PLD3* mRNA levels and pyrosequecing intergroup differences was assessed by *T* test. One-way analysis of variance (ANOVA) followed by Games-Howell *post hoc* analysis was used to analyze differences in the expression levels of *PLD3* mRNA between Braak and Braak stage groups. A logistic regression model (ENTER method) was fit to assess the independent association of *PLD3* mRNA levels with AD status, using gender and age as covariates. Kendall’s tau-b correlation coefficient was used to determine correlation between AD-related pathology and *PLD3* mRNA expression levels. Difference between two bisulfite cloning sequencing groups was evaluated with Mann-Whitney *U* test. GraphPad Prism version 6.00 for Windows (GraphPad Software, La Jolla, CA, USA) was used to draw graphs except for methylation figures that were obtained by QUMA software.

## Results

### *PLD3* expression is downregulated in Alzheimer’s disease hippocampus

**Fig. 1:**
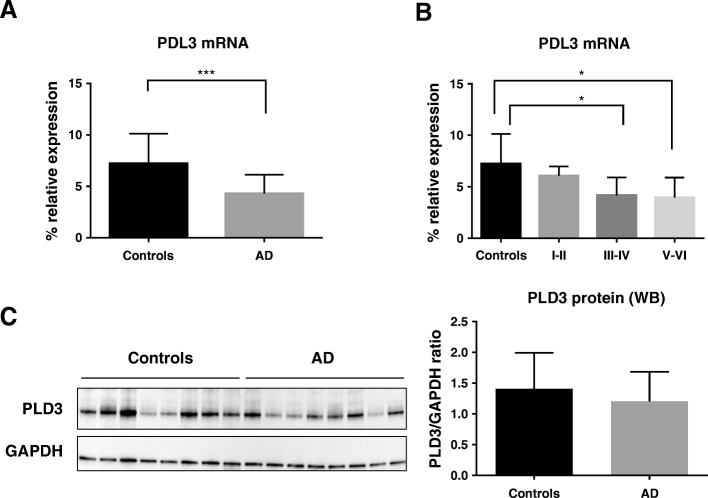
PLD3 expression is decreased in human hippocampus in Alzheimer’s disease (AD). **a** The graph shows a significant 1.67-fold decrease in *PLD3* mRNA levels in AD hippocampal samples compared to control hippocampal samples. **b** *PLD3* mRNA expression decreased across AD stages, as shown when *PLD3* mRNA expression levels are sorted by Braak and Braak stages. Bars represent percentage of *PLD3* mRNA expression relative to *ACTB* housekeeping gene expression. Vertical lines represent the standard error of the mean. *p value < 0.05; ***p value < 0.001. **c** Western blot analysis of PLD3 shows a mild protein expression decrease in AD. Human hippocampus samples from controls or AD patients were loaded as labeled on top of lanes. GADPH expression is shown as reference control. The bar chart represents the quantitative measurement of the PLD3 protein relative to GAPDH protein expression

As the first step in this study, we measured *PLD3* mRNA expression levels by real-time quantitative PCR (RT-qPCR) in the hippocampus of AD patients compared to controls. Five samples did not pass the RNA quality threshold (see the “Methods” section) and so were not included in the experiments. Eventually, 26 AD cases were compared to 11 controls. As shown in Fig. 1a, *PLD3* mRNA levels were significantly decreased by 1.67-fold in the hippocampus of AD cases compared to controls [*p* value < 0.001]. Next, a disease-staging analysis was performed to investigate changes of *PLD3* mRNA levels depending on the AD severity measured by Braak & Braak staging [21] . We found that *PLD3* mRNA levels were significantly reduced across Braak & Braak stages [*p* value < 0.005; Fig. 1b]. Games-Howell post hoc analysis revealed that *PLD3* mRNA expression was significantly different between control and Braak stages III–IV [*p* value < 0.05] and between control and Braak stages V–VI [*p* value < 0.05] (Fig. 1).

Then, to identify adjusted estimates of the association of *PLD3* mRNA levels with AD status (control = 0; AD = 1), a logistic regression model was designed. Age and gender were included into the model to adjust for potentially confounding variables. As shown in Table 1, *PLD3* mRNA expression levels remained as an independent predictor of AD status after adjusting for age and gender [*p* value < 0.05] (Table 1).

**Table 1:** Adjusted logistic regression model to predict AD status

Variable	B	Wald	*p* value	OR
*PLD3* mRNA levels	− 0.544	4.212	0.040*	0.581
Gender (female)	0.613	0.286	0.593	1.847
Age < 65 years old	2.774	5.981	0.014*	16.02
Constant	− 1.494	0.254	0.614	0.224

Alzheimer status (control = 0; AD = 1) was considered as the dependent variable and *PLD3* mRNA expression levels, gender, and age were included as covariates

*B* regression coefficient, *OR* odds ratio

*p value < 0.05

In order to examine whether the decrease in *PLD3* mRNA levels in the AD hippocampus extended to the protein level, a Western blot analysis was performed. Protein extracts from frozen hippocampal samples that were included in the qPCR experiment were obtained, and a polyclonal antibody against a recombinant protein epitope signature tag (PrEST) of PLD3 was used. GAPDH protein detection was used as housekeeping. In line with the *PLD3* mRNA expression results, we observed that PLD3 protein expression tends to be decreased in samples from hippocampus of AD patients as compared to controls (Fig. 1c).

### Correlation of *PLD3 mRNA* expression levels with p-tau and amyloid deposits

Next, we aimed to correlate *PLD3* mRNA levels with AD-related neuropathological changes in hippocampal sections. In brief, β-amyloid and hyperphosphorylated tau (p-tau) burden were measured and averaged for each subject by a semi-automated quantitative method by using the ImageJ software (see the “Methods” section). The amyloid plaque score (APS) was also recorded. As β-amyloid and p-tau data were not normally distributed, the non-parametric Kendall’s tau-b correlation coefficient was used. We found that the average area of β-amyloid burden in the hippocampus was inversely correlated with *PLD3* mRNA levels [tau-b = − 0,331; *p* value < 0.01], and accordingly, an inverse association was found between APS and *PLD3* mRNA levels [tau-b = − 0,319; *p* value < 0.01]. Regarding p-tau deposits, a statistically significant correlation was found for an inverse correlation [tau-b = − 0,306; *p* value < 0.01].

### DNA methylation in *PLD3* is increased in hippocampus of AD cases compared to controls

**Fig. 2:**
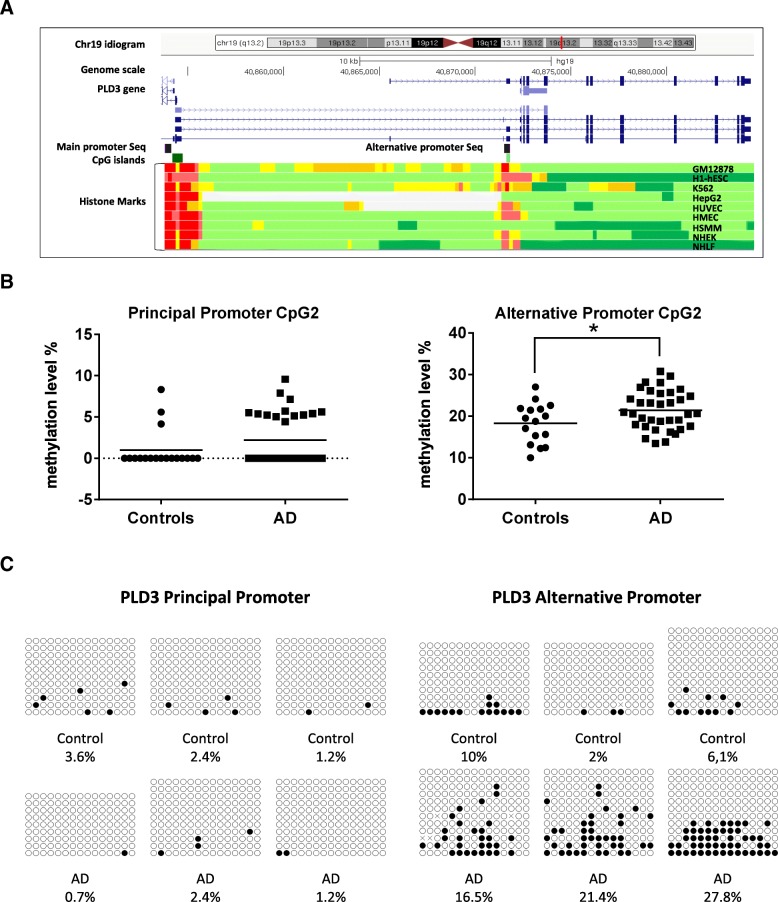
*PLD3* DNA methylation levels in human hippocampal samples. **a** The graph shows genomic position of the amplicons (black boxes) validated by bisulfite cloning sequencing which contain the cytosines assayed by pyrosequencing (CpG1 and CpG2) within the promoter regions (principal and alternative) of the *PLD3* gene. *PLD3* is located on the long arm of chromosome 19 (chr19:40,854,332-40,884,390 -GRchr19/hg19 coordinates). CpG islands are represented by isolated green boxes. At the bottom of the graph, predicted functional elements are shown for each of nine human cell lines explored by chromatine imunoprecipitation (ChIP) combined with massively parallel DNA sequencing. Boxes represent promoter regions (red), enhancers (yellow), transcriptional transition and elongation (dark green), and weak transcribed regions (light green). The track was obtained from the *Chromatin State Segmentation by HMM from ENCODE/Broad* track shown at the UCSC Genome Browser. **b** Dot-plot charts representing methylation levels for principal and alternative promoter of *PLD3* by pyrosequencing. Horizontal lines represent median methylation values for each group.*p value < 0.05. **c** Representative examples of bisulfite cloning sequencing validation for the two independent amplicons (principal and alternative promoter regions). Black and white circles denote methylated and unmethylated cytosines respectively. Each column symbolizes a unique CpG site in the examined amplicon, and each line represents an individual DNA clone

DNA methylation levels of regulatory regions in the genome modulate the expression of related or nearby genes. Thus, we tested whether DNA methylation levels in *PLD3* gene were also altered in the AD hippocampus. *PLD3* gene is located in the long arm of chromosome 19 (19q13.2) and has two distinct CpG island-containing promoter regions as shown by the UCSC Genome Browser website [25] (Fig. 2a). The principal promoter, which is placed at the 5′ end of the gene, contains a 553 bp CpG island (chr19:40854181-40854733; GRCh37/hg19) while an alternative promoter overlapping exon 2 contains a smaller 207 bp CpG island (chr19:40871618-40871824; GRCh37/hg19). Pyrosequencing primers were designed to amplify and sequence specific CpGs within both promoters regions (P_prom CpG1 and CpG2 for the principal promoter and A_prom CpG1 and CpG2 for the alternative promoter) (Fig. 2a).

We observed that the principal promoter of *PLD3* was mostly demethylated [mean ± SD, 1.8 ± 2.9%], as it corresponds to the constitutive promoter of an actively expressed gene. Average DNA methylation levels were slightly higher in AD cases compared to controls only for P_prom CpG2 [2.6 ± 3.13% vs. 0 ± 0%; *p* value < 0.001]. The alternative promoter showed intermediate levels of DNA methylation [20.5 ± 4.91%]. A_prom CpG1 showed a statistical trend to be highly methylated in AD cases compared to controls [23 ± 7.8% vs. 18.4 ± 6.2%; *p* value = 0.09] and a statistically significant difference in DNA methylation levels was observed for A_prom CpG2 in AD cases compared to controls [21.5 ± 5.8% vs. 15.3 ± 3.8%; *p* value < 0.01].

Next, we sought to replicate pyrosequencing results by extending the initial cohort with additional AD and control hippocampal samples for which DNA was available. These samples came from Navarrabiomed Brain Bank and were used to increase the sample size for the methylation experiments. Eventually, 36 AD patients and 18 controls were analyzed by pyrosequencing. In the principal promoter, average DNA methylation levels showed a trend to be higher in AD cases compared to controls at P_prom CpG2 [2.3 ± 2% vs. 1 ± 2.4% *p* value = 0.094]. In the alternative promoter, no differences were found for A_prom CpG1 between AD cases and controls [*p* value > 0.05]. However, we observed a statistically significant difference in DNA methylation levels for A_prom CpG2 between AD cases and controls [21.7 ± 4.7% vs. 18.3 ± 4.8%; *p* value < 0.05], pointing to a differentially methylated region located within the alternative promoter of *PLD3* in the AD hippocampus (Fig. 2b). In order to test whether A_prom CpG2 methylation was an independent predictor of AD status (control = 0; AD = 1), a binary logistic regression model was performed. After adjusting for age and gender, A_prom CpG2 methylation levels remain as an independent predictor of AD (Additional file 1: Table S3).

We validated the pyrosequencing results and extended the methylation local mapping by using bisulfite cloning sequencing in two independent amplicons overlapping both *PLD3* promoter regions. DNA methylation percentage was measured at CpG site resolution and further averaged across all the CpG sites for each amplicon. In line with the previous pyrosequencing results, we found that average DNA methylation levels of the amplicon at *PLD3* principal promoter were very low and showed no differences between AD patients and controls (Fig. 2c). On the contrary, average DNA methylation levels of the amplicon at *PLD3* alternative promoter were increased in AD patients compared to controls [19.1 ± 7.8% vs. 6 ± 4%; *p* value < 0.05] (Fig. 2c).

Since DNA methylation is one of the major mechanisms to regulate gene expression, we analyzed the correlation between *PLD3* mRNA expression and *PLD3* DNA methylation in our sample set. No significant correlation was found between expression and DNA methylation measured by pyrosequencing [A_prom CpG1 *r* = − 0.264, *p* value = 0.114; A_prom CpG2 *r* = − 0.275, *p* value = 0.110]. However, a significant inverse correlation was observed between expression and DNA methylation in the *PLD3* alternative promoter measured by bisulfite cloning sequencing [*r* = − 0.683; *p* value < 0.05].

## Discussion

We report *PLD3* gene to be downregulated at both transcript and protein level in the human hippocampus affected by AD. In addition, we show that the decrease in *PLD3* mRNA expression inversely correlates with β-amyloid burden in the hippocampus. An important finding of this study is that an alternative promoter of *PLD3* gene is differentially methylated in the hippocampus of AD patients compared to controls suggesting that epigenetic disturbances in *PLD3* may occur in the pathological process of AD.

Our results showing a reduction in *PLD3* expression in the AD hippocampus add to previous evidence supporting the idea that *PLD3* gene is downregulated in brain areas affected by AD processes [8, 9]. Cruchaga et al. [8] used data from genome-wide transcriptomics in laser-captured neurons from 33 AD cases and 16 controls (GEO dataset GSE5281) [26] to reveal that *PLD3* gene expression was significantly lower in AD cases compared to controls. In addition, Satoh et al. found a marginal reduction in *PLD3* mRNA levels in the frontal cortex of 7 AD cases compared to 14 non-AD subjects, including other neurodegenerative disorders such as amyotrophic lateral sclerosis and Parkinson disease [9]. In agreement with the previous results, we observed a statistically significant decrease in *PLD3* mRNA expression in the hippocampus, a vulnerable region to AD pathology, and also show that *PLD3* is reduced across Braak & Braak stages indicating that *PLD3* is somehow related to the progressive neurodegenerative processes of AD.

A number of different mechanisms could explain the decrease in *PLD3* gene expression in the AD hippocampus, including the progressive loss of neuronal populations, changes in cellular composition with increasing astrogliosis in late stages of AD, or cell-type-specific decrease in *PLD3* gene expression. A limitation of the present study is that it has been designed on a tissue-specific basis, and therefore, changes in gene expression at cell-specific level, including neuron-specific level, cannot be assessed. In fact, the ratio of cellular components in the human hippocampus may change across different stages of AD. In this case, and if the expression of *PLD3* were cell type specific, the gene expression changes observed globally in the hippocampus could be attributed to the loss of a given cell population and not reflect actual *PLD3* expression changes. However, the fact of having found epigenetic modifications in the same sample set would support the existence of a true alteration in the regulation of *PLD3* gene expression. To know whether the difference in *PLD3* gene expression is driven by a decreased expression in neurons or by changes in the ratio of cell populations in the brain of AD patients, other technologies, such as the emerging single-cell techniques, should be used.

The reduction in *PLD3* expression is in line with the classical β-amyloid cascade hypothesis of AD, since PLD3 protein seems to act as a negative regulator of APP processing [8]. It has been shown that knockdown of *PLD3* expression in cells results in higher levels of extracellular Aβ42 and Aβ40 levels, and conversely, overexpression of *PLD3* is associated with reduced extracellular Aβ42 and Aβ40 levels [8]. Furthermore, PLD3 protein is accumulated in neuritic plaques in human AD brains [9]. Indeed, it has been demonstrated that PLD3 protein can be co-immunoprecipitated with APP in cultured cells [8]. Even more, *PLD3* protein has been recently characterized as a novel endosome-to-Golgi retrieval gene that regulates the endosomal protein sorting, whose loss of function results in increased processing of APP [27]. Accordingly, we have found an inverse correlation between *PLD3* mRNA expression levels and the burden of hippocampal β-amyloid assessed by two measurements, averaged deposit of β-amyloid and amyloid plaque score (APS). All these data supports the notion that PLD3 protein could display a protective effect against AD pathology through its role in APP trafficking, as other authors have previously suggested [27].

Interestingly, PLD3 protein is co-expressed with other lysosomal proteins [9], including progranulin, which regulates lysosomal functioning and is also accumulated in neuritic plaques [9, 28]. Moreover, PLD3 protein is required to preserve the structure of lysosomes in vivo and, therefore, impairment of the endosomal-lysosomal systems has been proposed as an alternative mechanism by which PLD3 could contribute to the development of AD [29]. Most interestingly, another genetic variant in *PLD3*, p.Leu308Pro, was recently found to cause autosomal dominant spinocerebellar ataxia [30], a neurodegenerative condition where lysosomal disturbances are thought to be crucial [31, 32]. As an additional alternative explanation, PLD3 might also influence AD pathological processes by altering adult neurogenesis since *PLD3* gene expression seems to be turned on at late stages of neurogenesis [33].

Finally, we describe an altered pattern of DNA methylation within an alternative promoter of the *PLD3* gene in the human hippocampus affected by AD. To our knowledge, no previous reports on altered DNA methylation in *PLD3* gene have been published and very little is known about regulation of *PLD3* gene expression. The alternative promoter of *PLD3* is placed ∼17,500 bp downstream the principal promoter overlapping exon 2. It contains a small CpG island and is conserved across several cell types (Fig. 2a). In our study, it was found to be differentially methylated showing higher DNA methylation levels in AD patients than in controls. Since DNA methylation of CpG islands is one of the major epigenetic mechanisms that influence gene expression, our results indicate that altered DNA methylation at this particular regulatory region might contribute to downregulate *PLD3* expression in AD.

In this regard, we also show a significant correlation between *PLD3* mRNA expression and DNA methylation in our dataset when measured by bisulfite cloning sequencing, while the pyrosequencing results did not show correlation with expression. It is intriguing why the significant correlation is found only for the bisulfite cloning sequencing results. First of all, although not significant, an inverse correlation appears in the statistical analysis for the pyrosequencing results. However, it is only a statistical trend. One possible explanation would be that DNA methylation levels measured by bisulfite cloning sequencing average the methylation levels of an extended genomic region (15 CpGs), and therefore, this result may be more close to the real functional effect of methylation on gene expression than the result of individual CpGs.

Epigenetic disturbances are increasingly being described for a number of genes related to AD, including genes harboring rare variants that contribute to developing AD [34–40]. In this sense, our work provides new knowledge about the epigenetic alterations involved in gene transcription regulation in key brain regions for the development of AD. Additionally, our results support the involvement of *PLD3* in the pathology of AD.

## Conclusions

To sum up, this study confirms that *PLD3* gene is downregulated in the hippocampus of AD patients. Moreover, *PLD3* expression inversely correlates with β-amyloid burden, which adds evidence to the hypothesis that PLD3 protein may contribute to AD development through modifying APP processing. Having identified a differentially methylated region in an alternative promoter of *PLD3*, our study suggests that epigenetic disturbances in *PLD3* gene may be involved in the pathological processes of AD.

## Additional file

Additional file 1: **Table S1.** Brain sample set analyzed in our study. **Table S2.** RT-qPCR and bisulfite PCR primers. **Table S3.** Adjusted logistic regression model to predict AD status. (PDF 465 kb)

## Abbreviations

AD: Alzheimer’s disease
ANOVA: Analysis of variance
APOE4: ε4 allele of the *APOE* gene
APP: Amyloid precursor protein
APS: Amyloid plaque score
cDNA: Complementary DNA
CpG: Cytosine-phosphate-guanine dinucleotide
GEO: Gene Expression Omnibus
*PLD3*: Phospholipase D family, member 3
PrEST: Protein epitope signature tag
p-tau: Hyperphosphorylated tau
QQ: Quantil-quantil plots
RT-qPCR: Real-time quantitative PCR
Tau AT8: Mouse monoclonal antibody anti-human PHF-TAU, clone AT-8
UCSC: University of California, Santa Cruz
V232M: Val232-to-met substitution
