# Supplementary material for: PLD3 epigenetic changes in the hippocampus of Alzheimer’s disease
Source: Clin Epigenetics. 2018 Sep 12;10:116. doi: 10.1186/s13148-018-0547-3 (PMC6134774; doi:10.1186/s13148-018-0547-3)
Supplement: Supplementary file 1 — Table S1. Brain sample set analyzed in our study. Table S2. RT-qPCR and bisulfite PCR primers. Table S3. Adjusted logistic regression model to predict AD status. (PDF 465 kb) [file 13148_2018_547_MOESM1_ESM.pdf]

Additional File 1

Supplemental Table S1. Brain sample set analyzed in our study.

No.	Diagnosis	Braak stage	APS	Age at death (years)	Gender	PMI (h)	Cohort type
1	Control	0	NA	61	male	8	original
2	Control	0	NA	81	male	10,5	original
3	Control	0	NA	43	female	3	original
4	Control	0	NA	88	male	9	original
5	Control	0	NA	53	male	7	original
6	Control	0	NA	41	male	3,5	original
7	Control	0	NA	28	male	6	original
8	Control	0	NA	46	female	7	original
9	Control	0	NA	69	male	12	original
10	Control	0	NA	19	female	NA	original
11	Control	0	NA	26	male	6,2	original
12	Control	0	NA	54	male	18	original
13	Control	0	NA	54	male	2,7	additional
14	Control	0	NA	66	male	6,5	additional
15	Control	0	NA	88	female	3,5	additional
16	Control	0	NA	76	female	11,5	additional
17	Control	0	NA	65	male	3	additional
18	Control	0	NA	69	female	15	additional
19	AD	I	0,00	60	male	15,3	original
20	AD	I	2,00	85	male	3,2	original
21	AD	I	NA	85	female	2	additional
22	AD	I	NA	80	male	3	additional
23	AD	II	0,00	66	female	1,4	original
24	AD	II	NA	80	female	3,7	additional
25	AD	II	NA	71	female	4	additional
26	AD	II	NA	71	male	11,5	additional
27	AD	II	NA	74	male	2,5	additional
28	AD	III	0	85	female	4,3	original
29	AD	III	0,00	88	female	33	original
30	AD	III	3,00	96	female	1,5	original
31	AD	III	0,33	79	female	13	original
32	AD	III	2,33	84	female	13	original
33	AD	III	2,00	98	female	23	original
34	AD	III	2,67	85	female	NA	original
35	AD	III	3,67	83	male	9	original
36	AD	III	6,67	69	female	4,3	original
37	AD	III-IV	1,00	81	female	9	original
38	AD	III-IV	6,67	98	female	3	original
39	AD	IV	5,00	88	male	3,5	original

40	AD	IV	2,33	91	female	10	original
41	AD	IV	1,33	84	male	3,3	original
42	AD	IV	3,00	97	female	NA	original
43	AD	IV	1,33	78	male	5	original
44	AD	IV	1,33	90	female	3	original
45	AD	V	3,00	92	female	14	original
46	AD	V	4,00	77	female	11	original
47	AD	V	7,00	82	female	9	original
48	AD	V	8,00	91	male	5	original
49	AD	V	5,67	77	female	4	original
50	AD	VI	3,33	93	female	3	original
51	AD	VI	8,00	86	female	2,3	original
52	AD	VI	4,33	61	male	10	original
53	AD	VI	9,67	70	male	2,35	original
54	AD	VI	8,33	59	male	4	original

The table shows the characteristic of the samples included in the study (n=54). No.: Number; APC: amyloid plaque score; h: hours; AD: Alzheimer's disease; PMI: *post mortem* interval; NA=not applicable.

Supplemental Table S2. RT-qPCR and Bisulfite PCR primers.

ID	Accesion number	Purpose	Amplicon Size	Tm	Primer F	Tm2	Primer R
PLD3 bis	NA	Bisulfite PCR	269	59.6	TTTTTTTTGTTTTTTATTTGTGGTTTT	59.09	AACCCCAACTTCTATCTAAACAAACTA
PLD3_prom bis			318	59.1	TGGGATTATTTGTTTTTATTGTATAGATG	59.37	ATTTCTTTAAACCTCAATTTCCTCC
PLD3 pyro	NA	Pyrosequencing	240	60.1	ATTTTAGGGTGGAATGTAGTAAAGT	61	AACTACCTCCCATAAACCCCAACTTCTA
PLD3 seq				40.7	GTGAGTTAGGATTGGG		
PLD3_prin pyro	NA	Pyrosequencing	112	55.5	ATGAGAAAGGAGTATGTTTGGT	58.3	CCCACACCCACCTTCCAAACTATA
PLD3_prin seq				42.1	TTGGTTTTAGTAGGTTTGTAGTTTA		
PLD3_q	NM_012268	qPCR	141	61.8	TTCTATGACACCCGCTACAAC	62.1	CACGTTGAGTAGAGCCTTCAG

The table shows the primer pairs used in the study. Amplified transcripts are identified by RefSeq Accession or GeneBank accesion number. qPCR: quantitative PCR; bp: base pair; Tm: Melting Temperature; NA: not applicable.

Supplemental Table S3. Adjusted Logistic Regression Model to predict AD status.

Variable	B	Wald	p-value	OD	CI 95% for OD
**A_prom CpG2 methylation levels**	0.085	6.104	0.013*	0.92	0.86-0.98
**Gender (Female)**	-0.271	0.113	0.737	1.31	0.27-6.37
**Age <65 yo**	-2.671	10.027	0.002**	14.45	2.77-75.50

Alzheimer status (Control=0; AD=1) was considered as the dependent variable and PLD3 A_prom CpG2 methylation levels, gender and age were included as covariates. B: regression coefficient; OD: odds ratio; IC: confidence interval; yo: year-old; *: p-value <0.05; **: p-value <0.01
